# Algae Metabolites in Cosmeceutical: An Overview of Current Applications and Challenges

**DOI:** 10.3390/md18060323

**Published:** 2020-06-19

**Authors:** Krishnapriya Thiyagarasaiyar, Bey-Hing Goh, You-Jin Jeon, Yoon-Yen Yow

**Affiliations:** 1 Department of Biological Sciences, School of Science & Technology, Sunway University, Bandar Sunway 47500, Selangor Darul Ehsan, Malaysia; 15094311@imail.sunway.edu.my; 2 College of Pharmaceutical Sciences, Zhejiang University, 866 Yuhangtang Road, Hangzhou 310058, China; goh.bey.hing@monash.edu; 3 Biofunctional Molecule Exploratory (BMEX) Research Group, School of Pharmacy, Monash University Malaysia, Bandar Sunway 47500, Selangor Darul Ehsan, Malaysia; 4 Health and Well-Being Cluster, Global Asia in the 21st Century (GA21) Platform, Monash University Malaysia, Bandar Sunway 47500, Malaysia; 5 Department of Marine Life Sciences, Jeju National University, Jeju 63243, Korea; youjinj@jejunu.ac.kr

**Keywords:** marine algae, cosmeceuticals, UV-radiation, anti-aging, anticancer, skin whitening

## Abstract

Cosmetics are widely used by people around the world to protect the skin from external stimuli. Consumer preference towards natural cosmetic products has increased as the synthetic cosmetic products caused adverse side effects and resulted in low absorption rate due to the chemicals’ larger molecular size. The cosmetic industry uses the term “cosmeceutical”, referring to a cosmetic product that is claimed to have medicinal or drug-like benefits. Marine algae have gained tremendous attention in cosmeceuticals. They are one of the richest marine resources considered safe and possessed negligible cytotoxicity effects on humans. Marine algae are rich in bioactive substances that have shown to exhibit strong benefits to the skin, particularly in overcoming rashes, pigmentation, aging, and cancer. The current review provides a detailed survey of the literature on cosmeceutical potentials and applications of algae as skin whitening, anti-aging, anticancer, antioxidant, anti-inflammation, and antimicrobial agents. The biological functions of algae and the underlying mechanisms of all these activities are included in this review. In addition, the challenges of using algae in cosmeceutical applications, such as the effectiveness of different extraction methods and processing, quality assurance, and regulations concerning extracts of algae in this sector were also discussed.

## 1. Introduction

### 1.1. Synthetic Versus Natural Ingredients in Cosmetic Industry

Cosmeceuticals are topical cosmetic-pharmaceutical hybrids which refer to a cosmetic product with active ingredients claiming to have medicinal or drug-like benefits to skin health [1,2]. Globally, the cosmeceutical industry is growing each year due to the trend of modern lifestyle. More recently, the cosmeceutical industry is progressively shifting to natural bioactive ingredients because of the ineffectiveness of synthetic cosmetics [3].

Ineffectiveness of synthetic cosmetics includes their side effects and low absorption rate. The low absorption rate of cosmetics could be due to the big size of the molecular compounds. A study by Bos and Marcus [4] asserted that only compounds with the molecular weight lesser than 500 Dalton (Da) could penetrate through the skin. Cyclosporin (MW 1202 Da), a topical immunosuppressant, was not effective against psoriasis and allergic contact dermatitis as a higher molecular weight of the compounds inhibits skin penetration. Still, it was effective in psoriasis treatment when directly injected into the skin. Some of the side effects include irritation and allergic reaction to the users. According to a case study, hydroxybenzoic acid esters (parabens), which are widely used in cosmetic products, has been reported to mimic oestrogen; hence, increasing the incidence of breast cancer and causing the development of malignant melanoma [5].

In addition, a study on a population conducted by the Centers for Disease Control and Prevention reported that 97% of 2540 individuals were exposed to phthalates (a component of plastic that appears in cosmetic products; for instance, dibutyl phthalate in nail polish), which could result in DNA damage in human sperm [6]. In 2004, the Environment California, Environmental Working Group, and Friends of the Earth issued reports on cosmetic products containing chemical ingredients that lacked safety data. Some of these chemicals caused adverse effects in animal studies such as male genitalia congenital disabilities, altered pregnancy outcomes, and decreased in sperm counts [6]. As a result, consumers have changed their preference and opted for natural cosmetic products. The global market value for natural cosmetics was about $34.5 billion in 2018, and it is estimated to reach approximately $54.5 billion in 2027 [7]. The ever-expanding market for skincare products and continual search for innovative ingredients has led to the development of a multitude of cosmeceutical products based on natural bioactive ingredients, which include plants, herbs, and even marine algae [8].

Macroalgae are classified into three major classes, namely Phaeophyceae (brown algae), Rhodophyceae (red algae), and Chlorophyceae (green algae). Based on the total culture production, it is estimated that about 59% of brown algae, 40% of red algae, and less than 1% of green algae are produced worldwide [9]. Marine algae are rich sources of structurally diverse bioactive compounds, which are absent in other taxonomic groups. Algae contain 10 times greater diversity of compounds than terrestrial plants [10] and they have a totally different flavonoid composition from vegetables and fruits. Macroalgae are a rich source of catechins and flavonols [11]. Furthermore, algae-derived phlorotannin possesses a unique structure, which is not found in terrestrial plants and this compound may constitute up to 25% of the dry weight of brown algae [11]. Algae produce a wide array of primary metabolites, such as unsaturated fatty acids, polysaccharides, vitamins, and essential amino acids [12,13]. Additionally, many research findings reported that secondary metabolites derived from algae such as fucoidan, fucoxanthin, sulphated polysaccharide, polyphenol and fucosterol were shown to possess anti-inflammation, antioxidant, anticancer, antibacterial and anti-aging effects [14,15,16,17,18]. The demand for algae bioactive compounds in cosmeceuticals is rapidly increasing as they contain natural extracts which are considered safe; thus, rendering fewer side effects on humans. In ancient times, marine algae were used as medicine to treat skin-related diseases, such as atopic dermatitis and matrix metalloproteinase (MMP) related disease [12]. In a nutshell, marine algae are a promising resource for the development of cosmeceuticals.

Marine algae can survive in harsh conditions (i.e., withstand heat, cold, ultra-violet radiation, salinity, and desiccation) [8,9,19] due to their ability to adapt to physiological changes by producing stress tolerant substances. For example, algae produce organic osmolytes during stress conditions, which also act as antioxidants and heat protectants. Algae grow under desiccation by producing specialized spores which remain dormant during stress conditions and revive once the conditions return to normal. The presence of thick cell walls with protective layers of chemical substances and mucilage sheath helps to delay the process of desiccation. Algae that grow in cold desserts can endure the subzero temperature and protect the cells from UV irradiation by producing spores that have thick cell walls and reserve food as lipid and sugars [20]. In addition, marine algae uptake inorganic ions to balance extracellular ion concentration and produce organic osmolytes which protects them from desiccation and UV lights. A study reported that *Dunaliella salina* has 55 novel membrane-associated proteins that showed changes in the composition and structure of the membranes associated with algae adaptation to salinity [21]. Algae are rich in a wide variety of secondary metabolites to help them adapt and survive in harsh conditions. Algae could also adapt to desiccation stress by producing specialized spores such as aplanspores, which are rich in astaxanthin. Astaxanthin is a carotenoid that protects the cells from photo-oxidation. Algae exposed to UV radiations will produce UV screening compounds such as mycosporine-like amino acids (MAA), which acted as antioxidants and involved in osmotic regulations. Furthermore, algae exposed to high solar radiation and low nitrogen concentration produce more β-carotene, such as *Dunaliella* [20]. Thus, algae that are naturally exposed to oxidative stress develop defense systems that protect them against reactive oxygen species (ROS) and free radicals. These compounds could be used in cosmetics to protect the cells against the adverse effects of UV radiation. Some of the environmental benefits of algae include fixation of carbon dioxide. Studies have reported that large cultivation of microalgae capable of uptaking carbon from the atmosphere; for instance, *Spirulina platensis* with carbon fixation rate of 318 mg/L^−1^d^−1^ and *Chlorella vulgaris* with carbon fixation rate of 251 mg/L^−1^d^−1^ [22,23].

The exploitation of marine algae for the environmental and industrial production of natural products is a fast-growing sector. These natural products are expected to become very competitive in the market due to their higher biological value, improvement in the cultivation process, and lower production cost than synthetic products.

### 1.2. Current Applications of Algae-Derived Metabolites in Cosmeceutical Industrial

The transition from synthetic compounds to natural products such as marine algae have been attracting the attention of many researchers since algae possess a wide range of pharmacological activities with negligible cytotoxicity effects in human cells [24]. Marine algae are used for different purposes in food, pharmaceutical, biofuel, agriculture, and cosmetic industries. Industries, such as Cyanotech, Fuji Chemical Seambiotic, and Mera Pharmaceuticals are producers of microalgae biomass contributing to products in pharmaceuticals, cosmetics, and nutritious feed [25]. Interestingly, phycocyanin (usually found in red algae and cyanobacteria) is accepted as a natural color additive in food and cosmetics by the Food and Drug Administration (FDA) due to its non-toxic, natural, and biodegradable properties. Accordingly, it becomes the major target of the market in the United States [26].

Meanwhile, carotenoid such as astaxanthin plays a crucial role in scavenging free radicals in the human body and it is considered a strong antioxidant; hence, its popularity as a human dietary supplement. Leading cosmeceutical industries, such as Unilever, L’Oreal, Henkel, and Beiersdorf are expected to improve the growth of carotenoid market value in the European market [27]. The market value for carotenoids is expected to reach about $1.53 billion by 2021 [27,28].

Furthermore, red algae *Gracilaria* account for most of the raw material for the agar extraction. It is reported by the Food and Agriculture Organization (FAO) of the United Nations that more than 80% of the agar were produced from *Gracilaria* species, which are mainly produced by China and Indonesia [29]. *Gracilaria* species have been widely used in cosmetics due to their stabilizing, thickening, and gelling characters. Commercially available products from *Gracilaria* species include hydrogel soap by Sea Laria^®^, facial mask by Balinique^®^, and hydrating cream by Thalasso^®^ [29].

A number of algae-based skin products have been marketed, such as Algenist (an anti-aging moisturizer containing microalgae oil and alguronic acid from algae) [30], Helionori^®^ by Gelyma and Helioguard365^®^ (a sunscreen product containing MAAs from red seaweed *Porphyra umbilicalis*) [31], Protulines^®^ by Exsymol S.A.M., Monaco (an anti-aging agent from protein-rich extract of *Arthrospira*), and Dermochlorella by Codif, St. Malo, France (an anti-wrinkling agent from *Chlorella vulgaris* extract) [32]. Therefore, bioactive compounds derived from algae could be considered a potential cosmeceutical agent for skincare.

### 1.3. UV Radiation and Skin-Related Diseases

Skin is one of the most complex and largest organs that serves as a protective barrier against water losses and environmental stresses, such as ultraviolet radiation (UVR), pathogens, physical agents, and chemicals [33]. The skin comprises three layers—epidermis, dermis, and hypodermis. The presence of keratinocyte cells and melanocyte cells in the epidermis layer plays a vital role in repairing damaged skin and protecting the skin from UV light. The dermis consists of elastin, hyaluronic acid, and collagen which involves tissue repair and stability, whereas hypodermis consists of fats, which involved in body insulation [9]. Several skin-related diseases that have been reported include acne, eczema, dermatitis, hives, psoriasis, and pityriasis rosea which cause rashes [34]. Other skin diseases include pigmentation disorders, such as hypopigmentation due to the absence of melanocytes and hyperpigmentation caused by a metabolic disorder or skin irritation. In addition, one of the biggest concerns is skin cancers (e.g., squamous, basal, and melanoma) with melanoma being the deadliest form in America because of overexposure to UV radiation [35].

**Figure 1:**
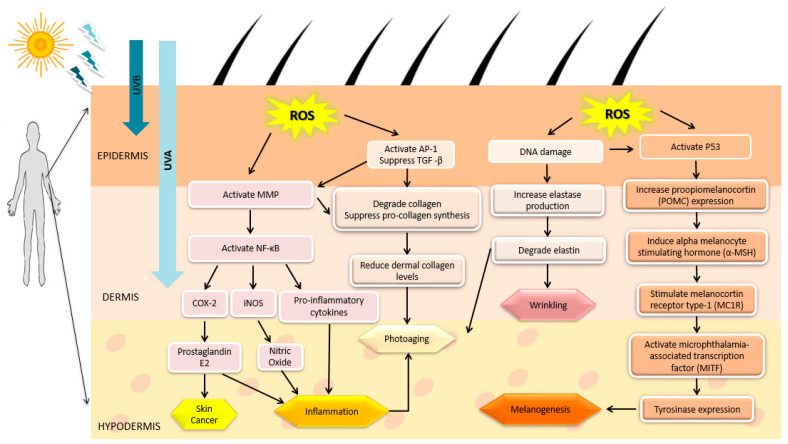
Effect of UV radiation-induced reactive oxygen species (ROS). Accumulation of ROS leads to skin cancer, inflammation, photoaging, wrinkling, and melanogenic through activation of respective signaling pathways.

In most cases, humans are exposed to UV radiation due to overexposure to sunlight. UV radiation can produce many adverse effects within the cells, including DNA damage, skin pathologies, such as erythema and inflammation, skin aging, and cancer [36]. There are three main components of UV radiation, namely UVA (315–400 nm), UVB (280–315 nm), and UVC (100–280 nm) [37]. UVA can reach the dermis layer of skin, increasing the level of ROS that indirectly induce DNA mutagenesis, which results in skin aging and wrinkling. UVA can act as a carcinogen by shortening telomere in the DNA strand and it has less ability to stimulate melanin production resulting in redness, sun tanning, and freckles. UVB can penetrate the epidermis layer and damage the DNA in skin cells directly and induce skin cancers. UVC is highly bioactive but humans are not exposed to UVC because it is mostly absorbed by the ozone layer. In addition, UV-induced oxidative stress plays a crucial role in causing aging, inflammation, melanogenesis and even cancer which are shown in Figure 1 [9,12,38,39,40,41].

## 2. Methods

We provide a comprehensive review of potential cosmeceutical compounds derived from 122 algae species. These compounds were searched by using keywords like ‘algae’, ‘seaweed’, ‘macroalgae’, or ‘microalgae’ combined with ‘cosmetic’ or ‘cosmeceutical’ in three major databases (ScienceDirect, PubMed, and Google Scholar).

**Figure 2:**
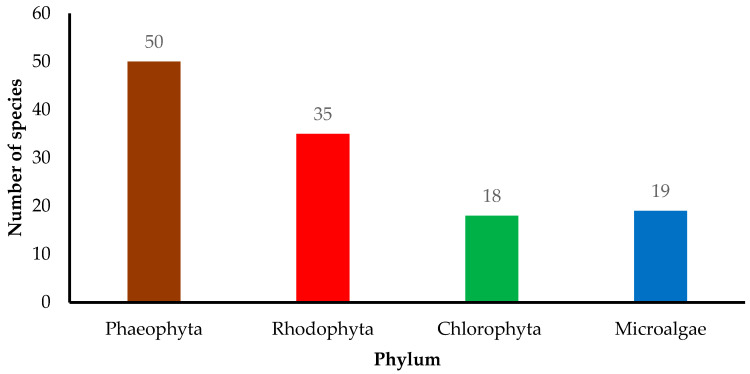
Distribution of algae species from different phyla in cosmeceuticals. The review was based on three major databases of ScienceDirect, PubMed, and Google Scholar.

**Figure 3:**
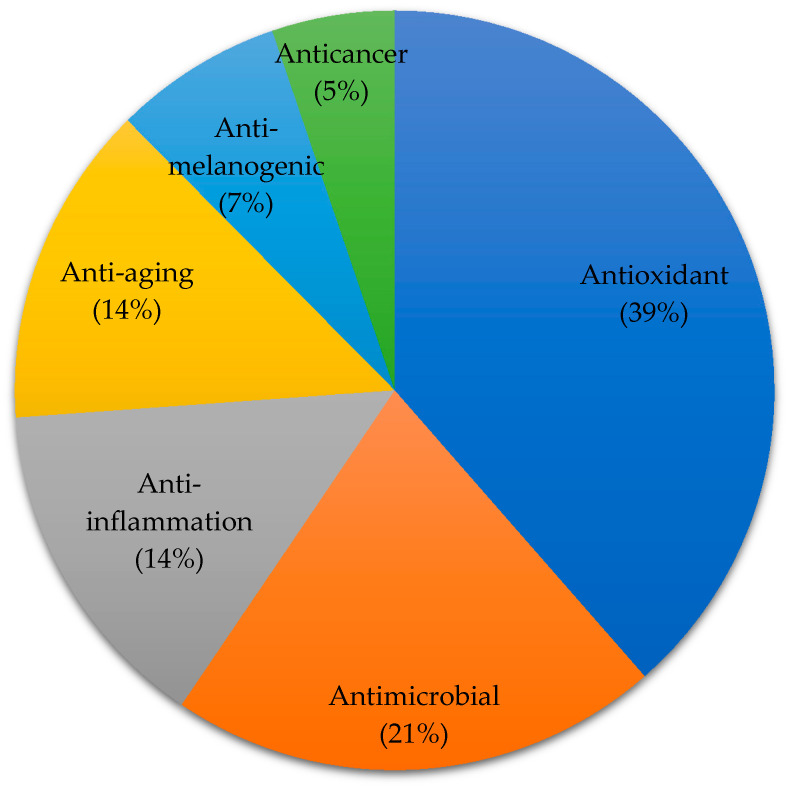
Distribution of algae-derived extracts or compounds with cosmeceutical properties. The result is based on 122 algae species (Some of the algae contribute to more than one biological functions).

Cosmeceutical properties of 50 Phaeophyta (brown), 35 Rhodophyta (red), 18 Chlorophyta (green), and 19 microalgae species are reported (Figure 2). In the present review, cosmeceutical properties of algae are classified into six activities, namely anti-aging (14%), antioxidant (39%), anti-inflammatory (14%), anti-melanogenic (7%), anticancer (5%), and antimicrobial (21%) (Figure 3). Some algae are reported to have multiple biological functions and they are counted as one.

## 3. Marine Algae-Derived Compounds in Cosmeceutical Application

**Figure 4:**
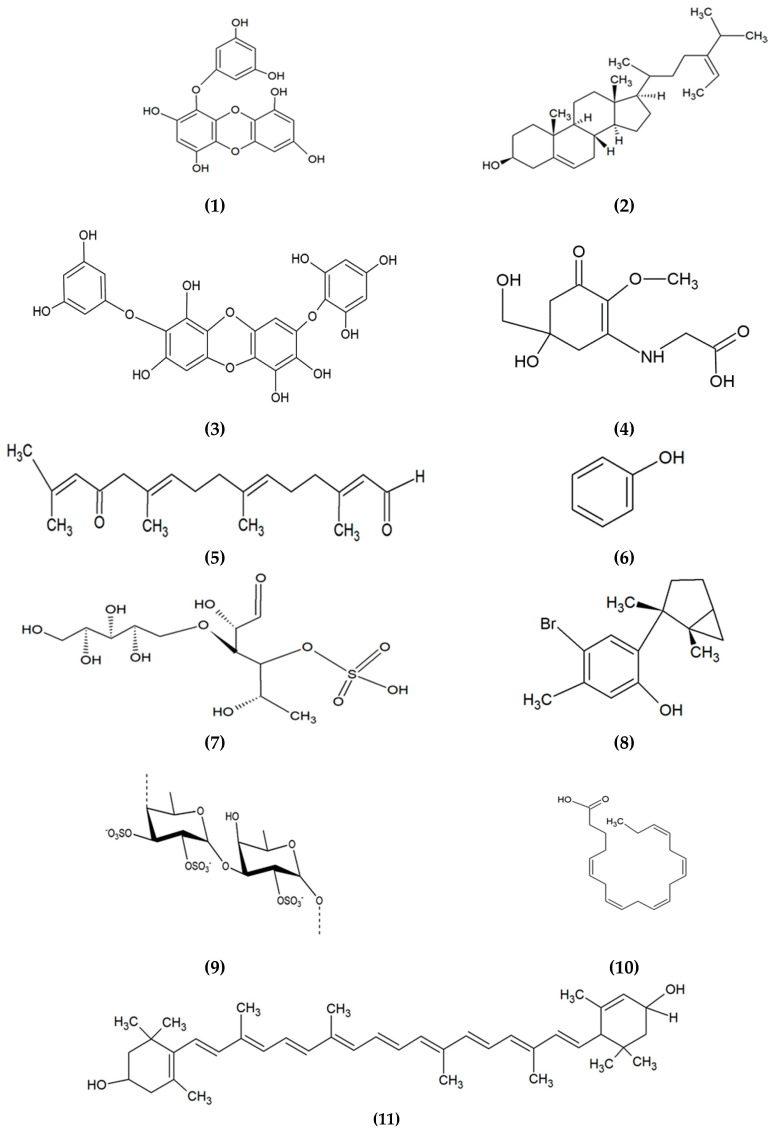

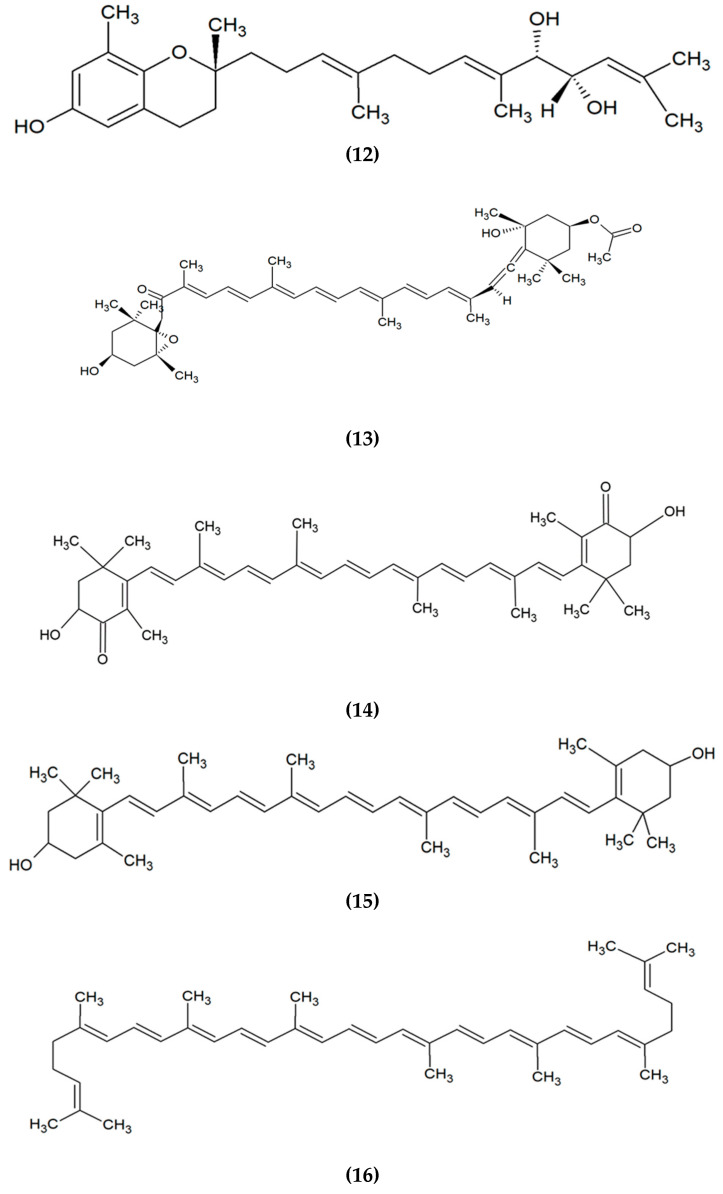
Chemical structures of bioactive compounds derived from algae. (**1**) Eckol, (**2**) Fucosterol, (**3**) Diphlorethohydroxycarmalol, (**4**) Mycosporine-glycine, (**5**) Eleganonal, (**6**) Phenol, (**7**) Ascophyllan, (**8**) Laurinterol, (**9**) Fucoidan, (**10**) Eicosapentaenoic acid, (**11**) Lutein, (**12**) Sargachromanol E, (**13**) Fucoxanthin, (**14**) Astaxanthin, (**15**) Zeaxanthin, and (**16**) Lycopene.

Based on the evidence from previous studies, brown algae contribute the most in cosmeceuticals. Some bioactive compounds from brown algae exhibit multiple cosmeceutical activities, including phlorotannin, which possesses several activities, such as anti-melanogenic, antioxidant, anti-inflammation, and anti-aging [12,42,43,44]. Likewise, fucoidan, a sulphated polysaccharide isolated from brown algae, contributes to anti-inflammation, anti-melanogenic and anticancer [45,46,47]. Fucoxanthin, a carotenoid isolated from brown, red, green and microalgae exhibit anti-melanogenic, anti-aging and antioxidant activities [48,49,50]. Mycosporine-like amino acids (MAAs), which are commonly found in red and green seaweeds, and microalgae also contribute to antioxidant, anti-inflammation, and anti-aging [51,52,53]. Other examples of bioactive compounds derived from algae, their applications and mode of actions in cosmeceuticals are presented in Table 1. The chemical structures of some prominent bioactive compounds are shown in Figure 4.

### 3.1. Anti-Aging Activity

Skin aging can occur through intrinsic and extrinsic mechanisms resulting in dryness, fragility, the formation of wrinkles, fine lines, laxity, and enlarged pores. Intrinsic mechanisms are due to genetic and physiological changes, whereas extrinsic mechanisms are caused by exposure to pollution, UVR, and infectious agents [97]. Extrinsic mechanisms result in the alteration of DNA, which leads to skin damage. UVA rays penetrate more deeply into the skin than UVB rays. Nevertheless, both can cause wrinkle formation or skin-related symptoms [33]. On the other hand, the intrinsic mechanisms involve the production of progerin, a lamin A protein that results in the stimulation of cellular senescence in normal human fibroblasts [132].

#### 3.1.1. Photo-Protectivity and Antioxidant Activities 

Damage to cellular components can be caused by ROS [97]. ROS is the initiator of oxidative stress, which include hydrogen peroxide, hydroxyl radicals, radical singlet oxygen, and superoxide anion radicals. It is known that UV radiation associated with the generation of ROS leads to the activation of a signaling pathway, such as MMP1-mediated aging, MAPK/AP-1/NF-κB/tumor necrosis factor (TNF-α)/IL-6-mediated inflammation-induced aging, and p53/BAX/cleaved caspase-3/cytochrome c-mediated apoptosis-induced aging [133]. ROS, that is generated by oxidative stress and can trigger apoptotic cell death, plays an essential role in intrinsic aging and extrinsic aging of the human skin, which eventually lead to skin cancer and inflammatory disorder [12,33,134]. Furthermore, lipid peroxidation of ROS causes the skin to lose its youthful appearance. The presence of antioxidant enzymes, such as catalase, superoxide dismutase, glutathione reductase, glutathione peroxidase, thioredoxin oxidase, and peroxiredoxin in skin cells protect them from UV-induced ROS and maintain the epidermal homeostasis [135]. Antioxidants protect skin from UV radiation by preventing membrane lipids, DNA, and protein damages caused by UV-induced ROS. Consequently, researchers are focusing on natural antioxidants derived from algae to avoid oxidation.

Natural antioxidants, such as carotenoid have anti-inflammatory and antioxidant activities that can protect the skin from UV rays and skin aging [136]. In addition, in vitro study on human dermal fibroblast cells using methanol extract of *Corallina pilulifera* showed that it has the ability to reduce UV-induced oxidative stress and expression of gelatinases. It is reported that the algae extract had successfully reduced the expression of UV-induced MMP-2 and MMP-9 in human dermal fibroblast and have antioxidant activity, which inhibits free radical oxidation [97]. This is due to the presence of phlorotannin (phenolic compound) that acts as MMP inhibitors [43]. Interestingly, microalgae, such as *Dunaliella salina* and *Spirulina platensis*, that is rich in β-carotene, and *Porphyridium*, which is rich in sulfated polysaccharides can prevent the formation of ROS [119], and inhibit lipid peroxidation [130] and inhibit oxidative damage, respectively [103]. Apart from that, the secondary metabolites of brown algae, *Macrocystis pyrifera* (i.e., phlorotannins) and *Turbinaria conoides* (i.e., laminarin, fucoidan, and alginate), act as an antioxidant agent, which prevents skin from aging [8,33]. Previous studies reported that *Laminaria ochroleuca* [74], *Porphyra haitanensis* [103], *Ulva pertusa*, *Enteromorpha linza*, and *Bryopsis plumose* possess antioxidant activity [108]. *Porphyra umbilicalis* (red alga) and *Chlorella sp.* (microalgae) contain MAAs; hence, acting as sunscreens [16,116]. Other examples include *Bifurcaria bifurcata* [55], *Cladosiphon okamuranus* [49]*, Cystoseira hakodatensis* [59], *Cystoseira barbata* [57], *Cystoseira foeniculacea* [58], *Ecklonia cava* [62], *Rhodella reticulata* [103], and *Undaria pinnatifida* [70] are algae species that have antioxidant activities.

Notably, zinc oxide (ZnO) and titanium dioxide (TiO_2_) are the only FDA-approved inorganic physical UV filters for sun protection to block UV radiation from penetrating the epidermis [137]. However, TiO_2_ in nano form in sunscreens that provide occlusive skincare can cause blackhead formation and photoallergic contact dermatitis [138]. It has been reported that cosmetics containing chemical sunscreen ingredients cause phototoxic and photoallergic [138]. Natural UV filters such as algae-derived UV filters from *Porphyra umbilicalis* prevent UV-induced DNA damage and inflammation, and *Codium fragile* protects against UVB-induced pro-inflammatory and oxidative stress [138,139].

#### 3.1.2. MMP Inhibition and Prevention of Collagen Degradation

The stimulation of mitogen-activated protein kinase by ROS causes phosphorylation of transcription factor activator protein 1, which results in the upregulation of MMP and eventually degrades skin collagen and leads to skin aging [97]. MMP is produced by keratinocytes, macrophages, fibroblasts, neutrophils, and mast cells. MMPs’ functional groups include interstitial collagenases that involve the degradation of Type I, Type II, and Type III collagen, gelatinases that involve the degradation of Type IV and Type V collagen, and stromelysin that degrades proteoglycan, laminin, and fibronectin [136]. UV-induced skin damage can be caused by the gelatinases, MMP-2 and MMP-9 as well. Collagen plays an essential role in maintaining the structural integrity and stability of tissues. The loss of structural protein like Type 1 collagen can result in the formation of wrinkles. Wrinkles are typically induced by intrinsic and extrinsic aging due to the upregulation of MMP-1 and elastase, which result in the breakdown of collagen and elastin [33]. Therefore, compounds with MMP, hyaluronidase and elastase inhibitory activities, and with collagen synthesis could be used in cosmetic products to prevent wrinkling on the skin.

Several studies have been conducted to determine the bioactive compounds in algae that can act as an anti-aging agent. Algae extracts from *Meristotheca dakarensis* and *Jania rubens* that produce glycosaminoglycans, Type I and Type III collagen synthesis, and keratin have been marketed [75]. A study on anti-aging by using *Pyropia yezoensis* (peptide PYP1-5) showed its ability to stimulate collagen synthesis, elastin synthesis, and suppress the expression of MMP-1 protein as well as enhance TGF-β1 protein, which involved in collagen synthesis [106]. Studies have found that the methanol extract of *Macrocystis pyrifera* contained hyaluronic acid and enhanced the production of syndecan-4, a protein component of the extracellular matrix and useful in anti-aging product [75]. Other examples include *Fucus vesiculosus* [8], *Hizikia fusiformis* [18], *Sargassum muticum* [85], *Chlorella vulgaris* [117], *Haematococcus pluvialis* [121], and *Undaria pinnatifida* [50], which showed that phytochemicals in marine algae are potential anti-photoaging agents in cosmetics.

According to a study by Joe et al. [43], *Ecklonia stolonifera* had a higher inhibitory effect on nuclear factor-kappa B (NF-κβ) and activator protein-1 (AP-1), which are the transcription factors involved in activating MMP-1 transcription, as compared to other seaweeds. The experiment was carried out by using reporter gene assay with reporter plasmids that contained NF-κB or AP-1. During the study, dieckol and eckol (phlorotannin) were extracted from *Ecklonia stolonifera* and tested on human dermal fibroblast. The results showed that these compounds inhibited the expression of MMP-1 by interfering with the expression of NF-κB and AP-1. These compounds are also involved in collagen synthesis, ROS inhibition, and cytokine blockade, whereby the findings provided evidence that phlorotannin can reduce the expression of MMP-1 and prevent skin aging.

A study by Kim et al. [83] reported that sargachromanol E derived from *Sargassum horneri* has been tested on UVA-irradiated dermal fibroblast, whereby it provides protection against UVA-induced collagen degradation by inhibiting MMP expression. It was found that the MMP expression was inhibited by tissue inhibitor of matrix metalloproteinase (TIMP)-1 and TIMP-2, which were treated with sargachromanol E. They demonstrated higher effect as compared to treatment with retinoic acid. It is reported that this compound has no risk of cytotoxicity as LPS-stimulated RAW 264.7 macrophages were still viable even when treated with a high concentration of the compound. Sargachromanol E can inhibit the formation of ROS and lipid peroxidation, and all these provide evidence that this compound is effective in preventing skin aging and could be a potential cosmetic agent.

#### 3.1.3. Anti-Inflammatory Activity

Inflammation is a non-specific response by our body to detect harmful stimuli that could damage the tissue or cause specific diseases. Mechanical injuries, chemical, biological, and physical agents as well as immunological disorders, can induce skin inflammation. Neutrophilic infiltration can damage chromatin and result in DNA mutations or promote intercellular transduction pathway, leading to inflammation. Inflammation is categorized into acute and chronic, whereby acute phase involves the fluid accumulation and an increase in blood flow, leukocyte, and vascular permeability, and chronic phase is associated with the initiation of the immune response [13].

The exposure of skin to UV radiation stimulates inflammatory responses, such as microvascular structural changes, vasodilation, transendothelial migration of leukocyte, and the escape of plasma protein [15]. UV radiation could trigger inflammation by inducing chemical reactions on the skin. The generation of high ROS during skin inflammation is to remove and destroy invading microorganism and to degrade damaged tissues [133]. The patterns of inflammation might vary based on exposure to a specific wavelength of light. UVB radiation is characterized as sunburn-induced erythema in the skin. UVB radiation induces inflammatory responses through mediators, such as nitric oxide, inducible NO synthase, prostaglandin E2, cyclooxygenase-2, tumor necrosis factor-α, and other cytokines, such as interleukin 1 and interleukin 6. These molecules produced in keratinocytes are regulated by NF-κB. NF-κB, which is associated with skin diseases, such as psoriasis, vulgaris, and allergic dermatitis that cause skin dryness, irritation, itching, swelling, redness, and rashes on the affected area [140] as well as induced MMP-1 expression, leading to aging. NF-κB is vital as it regulates telomerase gene expression, inflammation, cellular proliferation, angiogenic, anti-apoptotic, and it is responsible for cellular longevity [133]. Studies reported that *Ecklonia kurome* and *Ecklonia cava* extracts have anti-inflammatory effects by inhibiting nitric oxide production [42,61]. Furthermore, *Spirulina platensis* and *Dunaliella salina* inhibit nitric oxide synthesis [120,130] and *Porphyridium* sp. inhibits pro-inflammatory modulator [103]. Other examples of algae that have anti-inflammatory activities include *Eisenia bicyclis* [42], *Ecklonia stolonifera* [63], *Ishige okamurae* [73], *Codium fragile* [110], *Chlamydomonas hedleyi* [52], *Chlorella vulgaris* [118], *Ulva lactuca* [75], and *Undaria pinnatifida* [92].

### 3.2. Anti-Melanogenic Activity

Exposure of skin cells to UV radiation results in the production of melanin by melanocytes, as a measure to protect the cells. Melanin is the matured form of melanosome, which moves to keratinocyte and degrades to induce skin tanning and melanization [9,46]. The use of tyrosinase inhibitors is one way to achieve skin hypopigmentation [12,141] as tyrosinase is a key enzyme for the synthesis of melanin, which determines the skin and hair color [136]. Tyrosinase catalyses involve two stages; hydroxylation of L-tyrosine to 3,4-dihydroxy-L-phenylalanine (DOPA) and oxidation 3,4-dihydroxy-L-phenylalanine (DOPA) into dopaquinone [142].

UV radiation excites endogenous chromophores that stimulate ROS and induce DNA damage. DNA damage causes the stabilization of p53, which increases the expression of proopiomelanocortin (POMC). POMC produces an α-melanocyte-stimulating hormone (α-MSH) that acts on the melanocortin 1 receptor (MC1R) of basal melanocytes. Polymorphic variants of MC1R produce the red hair/fair skin phenotype, which results in the inability to tan. Consequently, there is an increase of cAMP and the transcription of microphthalmia-associated transcription factor (MITF). This initiates the transcription of pigmentation genes which synthesizes and transports melanin [140].

Notably, hydroquinone is a skin whitening agent that has been banned from cosmetics due to mutagenicity and its adverse side effects, such as cataract, pigmented colloid milia, exogenous ochronosis, sclera, and nail pigmentation [3,143]. However, naturally occurring hydroquinone, such as arbutin, a hydroquinone glycoside, and aloesin, a C-glycosylated chromone, have been used in the cosmetic industry as a skin whitening agent because of their strong inhibition of tyrosinase enzyme, which is responsible for pigmentation and no side effects were reported [143].

Studies have shown that marine algae possess tyrosinase inhibitor activities, especially brown seaweed that is rich in phloroglucinol, which can chelate copper in this enzyme [12]. Apart from that, phlorotannin (7-phloroeckol) from *Ecklonia cava* and zeaxanthin from *Nannochloropsis oculata* have skin whitening activities by inhibiting tyrosinase [44,124]. Another bioactive compound, fucoxanthin from *Laminaria japonica*, has been treated on UVB-irradiated guinea pigs and melanogenesis in UVB irradiated mice. It was reported that this compound had reduced the tyrosinase activity. Topical treatment of fucoxanthin on mice has down-regulated the mRNA expression of endothelin receptor A, p75 neurotrophin receptor, melanocortin 1 receptor (MC1R), and prostaglandin E receptor 1, resulting in the suppression of cyclooxygenase (COX)-2 expression, which interferes with prostaglandin in the epidermis [48]. The study reported that fucoxanthin suppressed the tyrosinase mRNA expression. However, the suppression was not significant. Interestingly, they found that fucoxanthin suppressed tyrosinase-related protein 1 (TRP1) instead of tyrosinase. They also demonstrated that the pigmentation in the guinea pigs was suppressed by a daily consummation of fucoxanthin of about 0.001% through their diet [39,48]. Furthermore, the oral treatment of fucoxanthin can inhibit transcription for melanogenesis by repressing mRNA expression of COX-2, p75NTR, EP1, and MC1R [39]. The findings showed that fucoxanthin has anti-pigmentary effect by suppressing prostaglandin E2 synthesis and melanogenic stimulant receptors [39,48].

Other algae species that possess anti-melanogenic activity include *Fucus vesiculosus* [46], *Hizikia fusiformis* [69], *Ishige foliacea* [71,72], *Petalonia binghamiae* [78], *Sargassum polycystum*, *Schizymenia dubyi* [39], *Undaria pinnatifida* [91]. The evidence indicated that bioactive compounds from seaweeds have great potential to be used as skin whitening and depigmentation agents. Therefore, researchers are paying great attention to marine algae to develop natural tyrosine inhibitors.

### 3.3. Anticancer Activity

Skin cancer is categorized into basal cell carcinoma, melanoma, and squamous cell carcinoma. Melanoma is the most common skin cancer, which is derived from melanocytes. Melanoma appears in brown, black, pink, or red shades and induces itchiness as well as bleeding. One of the common factors that lead to skin cancer is prolonged exposure to UV radiation [16]. UV radiation can trigger DNA mutations, such as converting cytosine to thymine that results in the formation of dimers. These mutations affect the functions of oncogene (bcl-2) and tumor suppressor gene (p53), an important housekeeping gene, and causes the cell cycle to be out of control; thus, transformed keratinocyte and melanocyte. This expression leads to the development of precursor lesions such as actinic keratosis, and the progression of this expression eventually results in squamous cell carcinoma, basal cell carcinoma, or melanoma by the accumulation of additional genetical alterations [144]. UV radiation can also alter signal transduction that affects mutation indirectly; for instance, by reducing the time taken for DNA repair or reducing the level of the enzyme that protects the cells from UV damage [144]. Accordingly, UV radiation is one of the common factors that cause skin cancer. The use of sunscreen, which could decrease the exposure to UV radiation is an effective method to prevent skin cancer. Previous studies reported that *Ascophyllum nodosum* [54], *Fucus evanesens* [45], *Stoechospermum marginatum* [89], *Porphyra yezoensis* [51], *Haematococcus pluvialis* [122], and *Skeletonema marinoi* [129] have anticancer activities, such as inhibiting cell proliferation, inducing apoptosis, preventing ROS production, reducing the expression of MMP, or by activating caspase.

A study by Hwang et al. [40] investigated the effect of dietary feeding and topical application of polyphenol extracted from brown algae on UVB radiation-induced skin carcinogenesis in SKH-1 mice. The results demonstrated that dietary feeding (0.1%) and topical application (3 mg) of polyphenol significantly reduced tumor multiplicity by 45% and 60%, respectively. Therefore, it was concluded that topical application of polyphenol gave a better impact on inhibiting skin cancer compared to dietary feeding. Interestingly, increasing the concentration of polyphenol in the topical application decreased its ability to reduce tumor multiplicity. The result showed that both dietary feeding and topical treatment of brown algal polyphenols, phlorotannin suppressed cyclooxygenase-2 (COX-2) expression and cell proliferation, which implies that polyphenol acts as an anticancer agent [12]. The study reported that UV radiation induces COX-2 expression and elevates the level of prostaglandin E2 (PGE2) [40]. PGE2 binds to G-protein receptors, such as EP1, EP2, and EP4, involves in tumor cell proliferation, inhibits apoptosis, facilitates immunosuppression and tumor invasions as well as stimulates the inflammatory response, which eventually induces skin cancer [145].

### 3.4. Antimicrobial Activity

Microbiological purity of cosmetics is one of the main problems faced by the cosmetic industry. Microbiological impurities increase the risk of infection to users and cause physicochemical changes in the cosmetic properties such as odor change, effectiveness, color and phase separation in solutions. In order to solve this issue, chemical compounds were added as preservatives to inhibit microorganism contamination and to maintain the physicochemical stability of the cosmetic products. However, the presence of synthetic preservatives (parabens, benzyl alcohol, and tetrasodium ethylenediaminetetraacetic acid) causes allergies to users; hence, natural preservatives are in demand to replace synthetic preservatives [146,147].

Some preservatives were considered toxic; therefore, the application and selection are strictly regulated by law and regulations in many countries [148]. Algae extracts are the promising preservative agents that can be used in cosmetics to prevent microbial, environmental, and personal contamination. In particular, the phenolic compound that improves the shelf-life of cosmetic products by inhibiting the growth of microorganisms [149]. In addition, antibacterial compounds derived from macroalgae can fight acne and chronic wounds. Kamei et al. [150] suggested that Sargafuran derived from *Sargassum macrocarpum* that showed antibacterial activity against *Propionibacterium acnes* might be useful as a lead compound for developing new skincare cosmetics to prevent acne.

The bioactive compound from algae such as phlorotannin contributes to antibacterial activity by inhibiting oxidative phosphorylation and binding to bacterial proteins, such as enzyme and cell membrane, which lead to cell lysis [128]. Algal polysaccharides that contain glycoprotein-receptor on their surface bind with the bacterial cell wall, cytoplasmic membrane, and DNA which increase the permeability of the cytoplasmic membrane and cause protein leakage. In addition, fatty acids derived from algae act as electron transport chain and oxidative phosphorylation inhibitors in the bacterial cell membrane. Subsequently, they interfere with the adenosine triphosphate energy transfer and inhibit enzymes such as bacterial enoyl-acyl carrier protein reductase that is necessary for the synthesis of fatty acids within the bacterial cell. This eventually causes cell lysis.

*Rhodomela confervoides* and *Padina pavonica* from Algeria possessed fungi that inhibiting the effect on *Candida albicans* and *Mucor ramaniannus*. Another bioactive compound, laurinterol from *Laurenicia pacifica* has the antibacterial property to treat infection caused by *Staphylococcus aureus* [9]. Meanwhile, *Himanthalia elongate*, *Synechocystis* sp., and *Pterocladia capillacea* possess antimicrobial activities against *Escherichia coli* and *Staphylococcus aureus* [68,104]. Other examples include, *Chnoospora implexa*, *Colpomenia sinuosa*, *Cystoseira osmundacea*, *Dictyopteris delicatula*, *Hydroclathrus clathratus*, *Padina concrescens*, *Rosenvingea intrincata*, *Sargassum horridum* [56], *Alsidium corallinum*, *Ceramium rubrum*, *Chondrocanhus acicularis* [93], *Chondrus crispus* [96], *Corallina vancouverensis*, *Ganonema farinosum*, *Gelidium robustum* [56], *Laurenicia luzoensis, Laurenicia rigida* [12], *Cladophora sp.*, *Codium sp.*, *Ulva dactilifera* [56], *Planktochlorella nurekis* [128], and *Spirulina platensis* [131] also have antimicrobial activities that can be used as a source of natural preservatives in cosmetic formulations.

## 4. Challenges of Algae in Cosmeceuticals

A study conducted by Klaschka [151] reported that there were 1358 natural substances listed in the International Nomenclature of Cosmetic Ingredients (INCI), of which, 369 were considered hazardous substances (i.e., 257 to the skin, 182 to the aquatic environment, and 53 with carcinogenic property). Evidently, none of the products is 100% safe to be used in cosmetic products. However, consumers have changed their preference and more inclined towards natural products, as it is less harmful compared to chemicals-based cosmetics. A diverse range of bioactive metabolites derived from marine algae has been given much importance in cosmetic products due to their multidirectional effects on the skin [9,12,136]. Algae are considered safe as they have negligible cytotoxicity effects when tested on human cell lines [24] and can be bioprocessed using eco-friendly novel extraction techniques [152]. They contain many bioactive compounds and fatty acids that are useful not only in the cosmetic sector, but also in biofuel production, wastewater treatment, CO_2_ sequestration, and oxygen release into the atmosphere, which eventually reduce the greenhouse effect [8]. In addition, algae are used in the food and pharmaceutical industry to develop food supplements for preventing neurodegenerative diseases [153]. By-products produced by these industries could also be a promising source of compounds with biological properties that are favorable for skin applications [149]. By doing these, algae could be an efficacious and cost-effective alternative to synthetic products. However, there are some challenges in using algae as a cosmeceutical ingredient, such as (i) biomass culturing techniques, (ii) metabolites extraction methods, and (iii) Quality assurance and regulations.

### 4.1. Biomass Culturing Techniques

Large scale algae cultivation requires optimum conditions, such as light intensity, pH control, free of contamination, nutrients availability, the presence of CO_2_, salinity, inorganic carbon, temperature, and nutrients [8,154]. In addition, the type of bioreactor used for algae cultivation is designed based on the type of algae and the purpose of culture. Algae are usually harvested from natural habitats (e.g., *Nostoc* sp. in Asia) or by cultivation under certain conditions [152]. Land cultivation near the sea is typically open cultivation such as tanks or ponds. Culturing algae in an open system is inexpensive, but it can be contaminated easily. Algae can be cultured by using seawater instead of freshwater, which enhances the production of lipids and other nutrients while reduces cost [154]. Nevertheless, this could be a challenge as well since open cultivation requires extensive use of seawater and the cost for the construction, operation and maintenance of the ponds or tanks is substantial. Open cultivation can cause interferences between algae cultures and the biotic and abiotic environments resulting in low quality of algae produced [155]. Closed cultivation of algae involves stirred tank reactors and closed bubble column photobioreactors for cultivating algal tissue [156]. Thus far, this method has not been able to produce large scale production. However, a study by Sebök et al. [157] designed a ring-shaped cultivation vessel that is better than cultivation tanks, as it can decrease the cost by reducing the usage of cultivation medium and eliminate the interference of the environment. Nevertheless, they reported that the algae initial growth rate is low until the optimal growth condition is determined.

### 4.2. Biometabolites Extraction Methods

Biometabolites extraction methods can be categorized into conventional and novel techniques. Conventional methods comprise saponification, maceration, soxhlet extraction, classical solvent extraction; whereas novel extraction methods include enzyme-assisted extraction (EAE), microwave-assisted extraction (MAE), supercritical fluid extraction (SFE), and ultrasound-assisted extraction (UAE) [152]. The novel techniques application is preferred as it offers several advantages including low extraction time, minimum usage of solvents, higher extraction yields, and quality.

#### 4.2.1. Enzyme-Assisted Extraction (EAE)

EAE is a safe and eco-friendly approach for extracting algae metabolites. EAE uses enzymes (e.g., peptidase, glucosidase, and carbohydrase) to disrupt the cell wall of algae to release the intracellular bioactive metabolites. This method can also remove the barriers of water solubility and insolubility of bioactive compounds, preserve the original efficacy of bioactive compounds and provide high catalytic efficiency [152,158]. Previous studies had proven that EAE is applicable for recovering different bio-compounds using selected enzymes, for instance, using alginase lyase enzyme to extract fucoxanthin from *Undaria pinnatifida*, and carbohydrases and proteases to extract antioxidants from *Sargassum horneri* [158,159]. The limitation of this method could be the cost of enzymes, lack of substrate-specific enzymes, and difficulty in maintaining bioreactor conditions [158]. Hyphenated extraction technologies can be adopted to overcome these limitations [160].

#### 4.2.2. Microwave-Assisted Extraction (MAE)

MAE can be operated in open and closed vessels. Open vessels are safer, effective, and able to process larger samples. MAE is environmentally friendly and economical because of the reduction of extraction time and minimization of the solvent consumption. Studies have reported that MAE has successfully extracted polyphenols from *Enteromorpha prolifera* [161] and polysaccharides from *Fucus vesiculosus* [162]. However, high power, temperature, and pressure tend to degrade and reduce the yield of the phenolic compounds. Low pressure exhibits less destruction on the structure of algae, resulting in a lower yield of polysaccharides released from the cells. In addition, an increase in time can increase the yield, but at the same time, excessive time can cause degradation of polysaccharide. MAE is a preferred method for extracting ulvan and rhamnan sulfate as the use of toxic solvents can be eliminated [163].

#### 4.2.3. Supercritical Fluid Extraction (SFE)

SFE uses carbon dioxide as a solvent because of its nontoxicity and low cost. Supercritical carbon dioxide (SC-CO_2_) extraction is applicable for extracting non-polar compounds, such as fatty acids, phenolic, phytosterols, triglycerides, tocopherols, and carotenoids [163]. According to past studies, this method isolated mostly phenolic compounds and carotenoid from *Cladophora glomerata, Ulva flexuosa, Chara fragilis,* and *Gracilaria mammillaris* [164]. Ethanol has been used as co-solvent to increase the extraction efficiency of phenolic compounds and carotenoids compared to the use of SC-CO_2_ alone [163,165].

#### 4.2.4. Ultrasound-Assisted Extraction (UAE)

UAE is usually used to extract phenolic compounds. The efficiency of UAE is influenced by temperature, time, and the power of an ultrasonic bath. Higher temperature facilitates in increasing the yield, and optimum extraction time is also required to prevent the degradation of phenolic compounds. UAE with acid as a solvent can extract laminarin from algae. One of the challenges faced by the UAE is cell disruption for the release of biomolecules [163]. The combination of two extraction techniques, such as maceration and ultrasonication, resulted in the highest yield and efficiency of extracting phycobiliproteins from *Gelidium pusillum* [166].

### 4.3. Quality Assurance and Regulations

Quality control and standardization of cosmetic products are crucial to ensure the safety, efficacy, and quality of products and its raw material. Some algae are high-value cosmetic raw materials, and it is essential to evaluate the presence of heavy metal like arsenic, mercury, lead and cadmium, pesticides, such as organochlorine, allergens, toxins, and other chemical contaminations in the algae samples. The level of contaminations must be low, as set by the World Health Organization and US Food and Drug Administration, for instance, lead and arsenic 10 ppm, mercury 1 ppm, and cadmium 0.3 ppm [152]. One of the precautions to be considered in developing cosmetic products is that certain drugs have side effects of phototoxicity. This is due to the presence of phototoxin or photoallergen, which is activated following skin contact and light exposure, known as phytophoto contact dermatitis. Many of these compounds are generators of singlet oxygen and other ROS [167]. Photoallergen such as chlorophyll can be categorized into four types, namely chlorophyll a in algae and cyanobacteria; chlorophyll b in green algae; chlorophyll c in brown algae, diatoms, dinoflagellates; and chlorophyll d in red algae [168]. Consumers are unaware that natural-based cosmetic products are made up of a complex mixture of natural raw materials and chemical compounds, which might develop potential adverse human health effects. Cyanotoxins, a metabolite derived from algae, has been reported to have toxic effects on the immune and brain cell; however, it could be used in agriculture as pesticides [169,170]. Bioaccumulation of algal toxins, such as aplysiatoxin, debromoaplysiatoxin, lingbyatoxin, and lipopolysaharide endotoxin, can also affect animal health [171]. Thus, a clinical study has to be carried out to determine the safety and efficacy of these compounds on human [151].

## 5. Conclusions and Future Studies

Cosmeceutical industries are exploring new compounds derived from natural products due to consumers’ demand, as synthetic cosmetic products are evidently causing adverse side effects on human. The current review focused on the importance of algae-derived compounds in cosmeceutical (e.g., antioxidant, anti-melanogenic, anticancer, anti-aging, antimicrobial, and anti-inflammation), scientific evidence of their commercial value, and their challenges and limitations. The current findings revealed that brown algae contributed the most in cosmeceuticals, followed by red algae, microalgae, and green algae. Furthermore, most of the algae possess antioxidant activities due to their ability to live in stress conditions. Interestingly, a diverse range of bioactive compounds, such as pigments, polysaccharides, phenol, lipids, and proteins have been isolated from algae for the cosmeceutical industry. In conclusion, bioactive compounds derived from algae possess antioxidant, anti-inflammation, anti-pigmentation, anti-aging, and anticancer activities; thus, they can be used as active ingredients in cosmetic formulations. Apart from that, natural compounds from algae are effective to be used in cosmeceuticals as they are less harmful to the skin compared to synthetic compounds. However, the exact mechanisms of these compounds in performing the biological functions have not been fully explored. Therefore, further studies are essential to understand the cell-signaling pathway and the exact mechanisms of the compounds. In order to improve the quality of cosmetic products, more clinical trials have to be carried out to determine skin absorption, irritation, genetic and phototoxicity, and allergens contents.

## Figures and Tables

**Table 1:** Bioactive compounds derived from algae and their applications in cosmeceuticals.

Algae Species	Bioactive Compound/Extract	Beneficial Activity	Mechanism of Action	Experimental Model	Reference
**Brown algae**					
***Ascophyllum nodosum***	Ascophyllan	Anticancer	Inhibit MMP expression	B16 melanoma cells	[54]
***Bifurcaria bifurcata***	Eleganonal	Antioxidant	DPPH inhibition	In vitro	[55]
***Chnoospora implexa***	Ethanol extract	Antimicrobial	Bacterial growth inhibition	*Staphylococcus aureus, Staphylococcus pyogenes*	[56]
***Chnoospora minima***	Fucoidan	Anti-inflammation	Inhibition of LPS-induced NO production, iNOS, COX-2, and PGE2 levels	RAW macrophages	[47]
***Cladosiphon okamuranus***	Fucoxanthin	Antioxidant	DPPH inhibition	In vitro	[49]
***Colpomenia sinuosa***	Ethanol extract	Antimicrobial	Bacterial growth inhibition	*S. aureus, S. pyogenes*	[56]
***Cystoseira barbata***	Fat-soluble vitamin and carotenoids	Antioxidant	High fat-soluble vitamin and carotenoid content	In vitro	[57]
***Cystoseira foeniculacea***	Polyphenol	Antioxidant	DPPH inhibition (EC_50_ = 5.27 mg/mL)	In vitro	[58]
***Cystoseira hakodatensis***	Phenol and fucoxanthin	Antioxidant	High total phenolic and fucoxanthin content	In vitro	[59]
***Cystoseira osmundacea***	Ethanol extract	Antimicrobial	Bacterial growth inhibition	*S. pyogenes*	[56]
***Dictyopteris delicatula***	Ethanol extract	Antimicrobial	Bacterial growth inhibition	*S. aureus, S. pyogenes*	[56]
***Dictyota dichotoma***	Algae extract	Antimicrobial	Inhibit the synthesis of the peptidoglycan layer of bacterial cell walls	*Penicillium purpurescens*, *Candida albicans*, *Aspergillus flavus*	[60]
***Ecklonia cava***	Dieckol	Anti-inflammation	Suppression of iNOS and COX-2	Murine BV2 microglia	[61]
	Phlorotannin	Anti-melanogenic	Inhibit melanin production	B16F10 melanoma cells	[44]
	Phlorotannin	Antioxidant	ROS scavenging potential	Chinese hamster lung fibroblast (V79-4)	[62]
***Ecklonia kurome***	Phlorotannin	Anti-inflammation	Inhibit hyaluronidase	Assay of HAase (In vitro)	[42]
***Ecklonia Stolonifera***	Phlorotannin	Anti-aging	Inhibit MMP expression	Human dermal fibroblast cell	[43]
	Phlorofucofuroeckol A and B	Anti-inflammation	Inhibition of NO production by downregulating iNOS and prostaglandin E2	LPS stimulated RAW 264.7 cells	[63]
***Eisenia arborea***	Phlorotannin	Anti-inflammation	Inhibit release of histamine	Rat basophile leukemia cells (RBL-2HE)	[64]
***Eisenia bicyclis***	Phlorotannin	Anti-inflammation	Inhibit hyaluronidase	Assay of HAase (In vitro)	[42]
***Fucus evanescens***	Fucoidan	Anticancer	Inhibit cell proliferation	Human malignant melanoma cells	[45]
***Fucus vesiculosus***	Extract	Anti-aging	Stimulate collagen production	N/A	[8]
	Fucoidan	Anti-melanogenic	Inhibit tyrosinase and melanin	B16 murine melanoma cells	[46]
	Fucoidan	Anticancer	Decrease melanoma growth	Mice	[65]
	Fucoxanthin	Antioxidant	Prevent oxidation formation	In vitro, RAW 264.7 macrophage, Mouse (ex vivo)	[66]
***Halopteris scoparia***	Ethanol extract	Anti-inflammation	COX-2 inhibition	COX inhibitory screening assay kit	[67]
***Himanthalia elongota***	Fatty acid andPhenol	Antimicrobial	Bacterial growth inhibition	*Escherichia coli, Staphylococcus aureus*	[68]
***Hizikia fusiformis***	Fucosterol	Anti-aging	Inhibit MMP expression	Human dermal fibroblast	[18]
	Ethyl acetate extract	Anti-melanogenic	Inhibit tyrosinase and melanin	B16F10 mouse melanoma cells	[69]
	Fucoxanthin	Antioxidant	DPPH inhibition	In vitro	[70]
***Hydroclathrus clathratus***	Ethanol extract	Antimicrobial	Bacterial growth inhibition	*S. aureus, S. pyogenes*	[56]
***Ishige foliacea***	Phlorotannin	Anti-melanogenic	Downregulation of tyrosinase and melanin synthesis	B16F10 cells Zebrafish embryo	[71,72]
***Ishige okamurae***	Diphlorethohydroxycarmalol	Anti-inflammation	Down-regulation of iNOS and COX-2 expression and NF-κβ activation	Human umbilical vein endothelial cells	[73]
***Laminaria japonica***	Fucoxanthin	Anti-melanogenic	Suppress tyrosinase activity	UVB- irradiated guinea pig	[48]
***Laminaria ochroleuca***	Polyphenol	Antioxidant	High total phenolic content and antioxidant capacity	In vitro	[74]
***Macrocystis pyrifera***	Phlorotannin	Antioxidant	ROS scavenging potential	In vitro	[8]
	Hyaluronic acid	Anti-aging	Enhance the production of syndecan-4	N/A	[75]
***Padina concrescens***	Ethanol extract	Antimicrobial	Bacterial growth inhibition	*S. aureus, S. pyogenes*	[56]
***Padina pavonica***	Polyphenol	Antimicrobial	Bacterial growth inhibition	*Candida albicans* and *Mucor ramaniannus*	[17]
	Acetone extract	Antioxidant	Free radical scavenging activity (IC_50_ = 691.56 µg L^−1^)	In vitro	[60]
***Padina tetrastromatic***	Diterpenes	Antioxidant	DPPH (IC_50_ = 1.73) & ABTS (IC_50_ = 2.01) inhibitions	In vitro	[76]
	Sulfated polysaccharide	Anti-inflammation	COX-2 and iNOS inhibitions	Paw edema in rats	[77]
***Petalonia binghamiae***	Ethanol extract	Anti-melanogenic	Inhibit tyrosinase and melanin	B16F10 murine melanoma cells	[78]
	Aqueous extract	Antioxidant Anti-inflammation	DPPH inhibition COX-2 inhibition	In vitro In vitro	[67]
***Rosenvingea intrincata***	Ethanol extract	Antimicrobial	Bacterial growth inhibition	*S. aureus, S. pyogenes*	[56]
***Saccharina latissima***	Phenol	Antioxidant	High total phenolic content, DPPH scavenging activity and FRAP	In vitro	[79]
***Sargassum fulvellum***	Fucoxanthin	Antioxidant	DPPH inhibition	In vitro	[70]
***Sargassum furcatum***	Methanol extract	Antioxidant	DPPH (EC_50_ = 0.461) & ABTS (EC_50_ = 0.266) inhibitions	In vitro	[80]
***Sargassum hemiphyllum***	Sulfated polysaccharide	Anti-inflammation	Inhibit LPS-induced inflammatory response	RAW 264.7 macrophage cells	[81]
***Sargassum henslowianum***	Sulfated polysaccharide	Anticancer	Activation of caspase-3	B16 melanoma cells	[82]
***Sargassum horridum***	Ethanol extract	Antimicrobial	Bacterial growth inhibition	*S. aureus, S. pyogenes*	[56]
***Sargassum horneri***	Sargachromanol.E	Anti-aging	Inhibit MMP expression	UVA irradiated dermal fibroblast	[83]
	Alginic acid	Anti-inflammation	Inhibit inflammatory response	HaCaT cells	[84]
***Sargassum muticum***	Tetraprenyltoluquinol chromane meroterpenoid	Anti-aging	ROS scavenging potential	Human dermal fibroblast	[85]
***Sargassum polycystum***	Ethanol extract	Anti-melanogenic	Inhibit tyrosinase and melanin production	B16F10 melanoma cells	[39]
***Sargassum serratifolium***	Sargachromenol	Anti-melanogenic	Downregulation of microphthalmia-associated transcription factor	B16F10 melanoma cells	[39]
***Sargassum siliquastrum***	Fucoxanthin	Antioxidant	Reduced UVB-induced ROS production	Human fibroblast	[86]
***Sargassum thunbergi***	Thunbergols	Antioxidant	DPPH inhibition	In vitro	[87]
***Sargassum vulgare***	Methanol extract	Antioxidant	β-carotene bleaching activity	In vitro	[88]
***Stoechospermum marginatum***	Spatane diterpenoids	Anticancer	Cell growth inhibition	Murine B16F10 melanoma cells	[89]
***Turbinaria conoides***	Laminarin, alginate, fucoidan	Antioxidant	ROS scavenging potential	N/A	[33]
***Turbinaria ornata***	Fucoxanthin	Antioxidant	High FRAP value (>10 µM/µg of extract)	In vitro	[90]
***Undaria pinnatifida***	Fucoxanthin	Anti-aging	MMP expression reduction, VEGF	Mouse	[50]
	Ethyl acetate extract	Anti-melanogenic	Down regulate melanin and inhibit tyrosinase	Mouse B16 melanoma cells	[91]
	Polyunsaturated fatty acid	Anti-inflammation	N/A	Mouse ear edema and erythema	[92]
	Fucoxanthin	Antioxidant	DPPH inhibition	In vitro	[70]
**Red algae**					
***Alsidium corallinum***	Methanol extract	Antimicrobial	Bacterial growth inhibition	*Escherichia coli, Klebsiella pneumoniae, Staphylococcus aureus*	[93]
***Bangia***	Algae extract	Antioxidant	Induce peroxidase and superoxide dismutase to reduce oxidative stress	In vitro	[94]
***Bryothamnion triquetrum***	Methanol extract	Antioxidant	DPPH (EC_50_ = 0.357) & ABTS (EC_50_ = 0.370) inhibitions	In vitro	[80]
***Ceramium rubrum***	Methanol extract	Antimicrobial	Bacterial growth inhibition	*Escherichia coli, Enterococcus faecalis, Staphylococcus aureus*	[93]
***Chondrocanthus acicularis***	Methanol extract	Antimicrobial	Bacterial growth inhibition	*E. coli, K. pneumoniae, E. faecalis, S. aureus*	[93]
***Chondrus canaliculatus***	Polysaccharide	Antioxidant	DPPH inhibition	In vitro	[95]
***Chondrus crispus***	Aqueous extract	Antimicrobial	Bacterial growth inhibition	*Salmonella Enteritidis*	[96]
***Corallina pilulifera***	Methanol extract	Anti-aging Antioxidant	Reduce the expression of gelatinase Inhibit free radical oxidation	Human dermal fibroblast Human fibrosarcoma (HT-1080)	[97]
***Corallina vancouverensis***	Ethanol extract	Antimicrobial	Bacterial growth inhibition	*S. aureus, S. pyogenes*	[56]
***Ganonema farinosum***	Ethanol extract	Antimicrobial	Bacterial growth inhibition	*S. aureus, S. pyogenes*	[56]
***Gelidium crinaale***	Fat-soluble vitamin and carotenoids	Antioxidant	High fat-soluble vitamin and carotenoid content	In vitro	[57]
***Gelidium robustum***	Ethanol extract	Antimicrobial	Bacterial growth inhibition	*S. aureus, S. pyogenes*	[56]
***Gracilaria gracilis***	Phenol	Antioxidant	ROS scavenging potential	In vitro	[98]
***Gracilariopsis lemaneiformis***	Sulfated polysaccharide	Antioxidant	DPPH, Superoxide radical assay, hydroxyl radical assay (EC_50_ = 2.45 mg/mL)	In vitro	[99]
***Gracilaria salicornia***	2H- chromenyl	Antioxidant Anti-inflammation	DPPH and ABTS inhibitions COX-1 inhibition	In vitro	[100]
***Jania rubens***	Glycosaminoglycan	Anti-aging	Collagen synthesis	Unknown	[75]
***Laurencia caspica***	Phenol Ethanol extract	Antioxidant Antimicrobial	DPPH inhibition Bacterial growth inhibition	In vitro *Klebsiella pneumonia, Pseudomonas aeroginosa*	[101]
***Laurencia luzonensis***	Sesquiterpenes	Antimicrobial	Bacterial growth inhibition	*Bacillus megaterium*	[12]
***Laurenicia obtusa***	Polysaccharide	Antioxidant	DPPH (IC_50_ = 24 µg/mL), FRAP (IC_50_ = 92 µg/mL), Hydroxyl radical scavenging activity (IC_50_ = 113 µg/mL)	In vitro	[102]
***Laurenicia pacifica***	Laurinterol	Antimicrobial	Bacterial growth inhibition	*Staphylococcus aureus*	[9]
***Laurencia rigida***	Sesquiterpenes	Antimicrobial	Bacterial growth inhibition	*Bacillus megaterium*	[12]
***Meristotheca dakarensis***	Glycosaminoglycan	Anti-aging	Collagen synthesis	Unknown	[75]
***Osmundaria obtusilo***	Methanol extract	Antioxidant	DPPH (EC_50_ = 0.041 mg/mL), ABTS (EC_50_ = 0.031 mg/mL), Metal chelating (EC_50_ = 0.1 mg/mL), folin ciocalteu (EC_50_ = 0.128 mg/mL)	In vitro	[80]
***Palisada flagellifera***	Methanol extract	Antioxidant	β-carotene bleaching activity	In vitro	[88]
***Palmaria palmata***	MAA	Anti-aging	Collagenase inhibition	*Clostridium histolyticum*	[53]
***Polysiphonia howei***	Fucoxanthin	Antioxidant	High FRAP value (>5 µM/µg of extract)	In vitro	[90]
***Porphyra haitanensis***	Sulfated Polysaccharide	Antioxidant	ROS scavenging potential	Mice	[103]
***Porphyra umbilicalis***	MAA	Anti-aging	Control expression of MMP	Human dermal fibroblast	[16]
***Porphyra* sp. **	MAA	Anti-aging	Collagenases inhibition	*Clostridium histolyticum*	[53]
***Porphyra yezoensis***	MAA Polyphenol Phycoerythrin	Antioxidant Anticancer Anti-inflammation	ROS scavenging potential and MMP expression Induce apoptosis Suppression of mast cells	Human skin fibroblast HaCaT cells Rat	[51]
***Pterocladia capillacea***	Sulfated polysaccharide	Antimicrobial	N/A	*Staphylococcus aureus* *Escherichia coli*	[104]
***Pyropia columbia***	Phenol	Antioxidant	DPPH, β-carotene bleaching and ABTS inhibitions	*Piaractus mesopotamicus*	[105]
***Pyropia yezoensis***	Polysaccharide	Anti-aging	Promote collagen synthesis	Human dermal fibroblast	[106]
***Rhodomela confervoides***	Polyphenol	Antimicrobial	Bacterial growth inhibition	*Candida albicans*, *Mucor ramaniannus*	[17]
	Bromophenol	Antioxidant	DPPH inhibition	In vitro	[107]
***Schizymenia dubyi***	Phenol	Anti-melanogenic	Inhibit tyrosinase activity	In vitro	[39]
***Green algae***					
***Bryopsis plumose***	Polysaccharide	Antioxidant	ROS scavenging potential	In vitro	[108]
***Chaetomorpha antennia***	Fucoxanthin	Antioxidant	DPPH inhibition (63.77%)	In vitro	[109]
***Chlamydomo-nas hedleyi***	MAA	Antioxidant Anti-aging Anti-inflammation	ROS scavenging potential Increase UV-suppressed genes (procollagen C proteinase enhancer and elastin) expression Reduce COX-2 and involucrin expression	In vitro HaCaT cells HaCaT cells	[52]
***Cladophora* sp. **	Ethanol extract	Antimicrobial	Bacterial growth inhibition	*S. aureus, S. pyogenes*	[56]
***Codium amplivesicula-tum***	Ethanol extract	Antimicrobial	Bacterial growth inhibition	*S. aureus, S. pyogenes*	[56]
***Codium cuneatum***	Ethanol extract	Antimicrobial	Bacterial growth inhibition	*S. aureus, S. pyogenes*	[56]
***Codium fragile***	Sterol	Anti-inflammation	Reduces the expression of COX-2, iNOS, and TNF-α	Mice	[110]
***Codium simulans***	Ethanol extract	Antimicrobial	Bacterial growth inhibition	*S. aureus, S. pyogenes*	[56]
***Entromorpha intestinalis***	Chloroform and methanol extract	Antioxidant	SOD activity is reduced	*Labidochromis caeruleus*	[111]
***Enteromorpha linza***	Polysaccharide	Antioxidant	ROS scavenging potential	In vitro	[108]
***Gayralia oxysperma***	Fucoxanthin	Antioxidant	High FRAP value (>6 µM/µg of extract)	In vitro	[90]
***Ulva dactilifera***	Ethanol extract	Antimicrobial	Bacterial growth inhibition	*S. aureus, Streptococcus pyogenes*	[56]
***Ulva fasciata***	Fucoxanthin	Antioxidant	DPPH inhibition (83.95%)	In vitro	[109]
***Ulva lactuca***	Phycocolloids	Anti-inflammation	N/A	N/A	[75]
***Ulva pertusa***	Polysaccharide	Antioxidant	ROS scavenging potential	In vitro	[108]
***Ulva prolifera***	Phenol and flavonoid	Antioxidant	DPPH inhibition, high phenolic and flavonoid contents	In vitro	[112]
***Ulva rigida***	Phenol	Antioxidant	DPPH inhibition	In vitro	[113]
***Ulva* sp. **	Sulfated polysaccharide	Anti-aging	Increase hyaluronan production	Human dermal fibroblast	[114]
***Microalgae/Cyanobacteria***					
***Anabaena vaginicola***	Lycopene	Antioxidant Anti-aging	N/A	In vitro	[115]
***Arthrospira platensis***	Methanol extracts of exopolysaccharides	Antioxidant	N/A	In vitro	[115]
***Chlorella fusca***	Sporopollenin	Anti-aging	Protect cells from UV radiation	N/A	[116]
***Chlorella minutissima***	MAA	Anti-aging	Protect cells from UV radiation	N/A	[116]
***Chlorella sorokiniana***	MAA	Anti-aging	Protect cells from UV radiation	N/A	[116]
	Lutein	Anti-aging	Reduce UV induced damage	N/A	[33]
***Chlorella vulgaris***	Hot water extract	Anti-aging	Reduced activity of SOD	Human diploid fibroblast	[117]
		Anti-inflammation	Downregulated mRNA expression levels of IL-4 and IFN-γ	NC/Nga mice	[118]
***Dunaliella salina***	β-carotene	Antioxidant	Protect against oxidative stress	Rat	[119]
	β-cryptoxanthin	Anti-inflammation	Reduced the production of IL-1*β*, IL-6, TNF-*α*, the protein expression of iNOS and COX-2	LPS-stimulated RAW 264.7 cells	[120]
***Haematococcus pluvialis***	Astaxanthin (carotenoid)	Anti-aging	Inhibit MMP expression	Mice and human dermal fibroblasts	[121]
		Anticancer	ROS scavenging potential	Mice	[122]
***Nannochloropsis granulata***	Carotenoid	Antioxidant	DPPH inhibition	In vitro	[123]
***Nannochloropsis oculata***	Zeaxanthin	Anti-melanogenic	Inhibit tyrosinase	In vitro	[124]
***Nitzschia* sp. **	Fucoxanthin	Antioxidant	Reduced oxidative stress	Human Glioma Cells	[125]
***Nostoc* sp. **	MAA	Antioxidant	ROS scavenging potential	In vitro	[126]
***Odontella aurita***	EPA	Antioxidant	Reduce oxidative stress	Rat	[127]
***Planktochlorella nurekis***	Fatty acid	Antimicrobial	Bacterial growth inhibition	*Campylobacter jejuni, E. coli, Salmonella enterica var.*	[128]
***Porphyridium* sp. **	Sulfated polysaccharide	Anti-inflammation Antioxidant	Inhibit proinflammatory modulator Inhibited oxidative damage	Unknown 3T3 cells	[103]
***Rhodella reticulata***	Sulfated polysaccharide	Antioxidant	ROS scavenging potential	In vitro	[103]
***Skeletonema marinoi***	Polyunsaturated aldehyde and fatty acid	Anticancer	Inhibit cell proliferation	Human melanoma cells (A2058)	[129]
***Spirulina platensis***	β-carotene and phycocyanin	Antioxidant Anti-inflammatio	Inhibit lipid peroxidation Inhibit TNF-α and IL-6 expressions	MouseHuman dermal fibroblast cells (CCD-986sk)	[130]
	Ethanol extract	Antimicrobial	Bacterial growth inhibition	*E. coli, Pseudomonas aeruginosa, Bacillus subtilis*, and *Aspergillus niger*	[131]
***Synechocystis* spp.**	Fatty acids and phenols	Antimicrobial	Bacterial growth inhibition	*E. coli S. aureus*	[68]
